# Early-onset atrial fibrillation patients show reduced left ventricular ejection fraction and increased atrial fibrosis

**DOI:** 10.1038/s41598-020-66671-w

**Published:** 2020-06-22

**Authors:** Laura Andreasen, Litten Bertelsen, Jonas Ghouse, Pia R. Lundegaard, Gustav Ahlberg, Lena Refsgaard, Torsten B. Rasmussen, Hans Eiskjær, Stig Haunsø, Niels Vejlstrup, Jesper H. Svendsen, Morten S. Olesen

**Affiliations:** 1 Laboratory for Molecular Cardiology, Department of Cardiology, Centre for Cardiac, Vascular, Pulmonary and Infectious Diseases, Rigshospitalet, University Hospital of Copenhagen, Copenhagen, Denmark; 2 Department of Biomedical Sciences, University of Copenhagen, Copenhagen, Denmark; 3 Department of Cardiology, Centre for Cardiac, Vascular, Pulmonary and Infectious Diseases, Rigshospitalet, University Hospital of Copenhagen, Copenhagen, Denmark; 4 Department of Cardiology, Aarhus University Hospital, Aarhus, Denmark; 5 Department of Clinical Medicine, University of Copenhagen, Copenhagen, Denmark

**Keywords:** Cardiovascular diseases, Cardiovascular genetics, Disease genetics

## Abstract

Atrial fibrillation (AF) has traditionally been considered an electrical heart disease. However, genetic studies have revealed that the structural architecture of the heart also play a significant role. We evaluated the functional and structural consequences of harboring a titin-truncating variant (TTNtv) in AF patients, using cardiac magnetic resonance (CMR). Seventeen early-onset AF cases carrying a TTNtv, were matched 1:1 with non-AF controls and a replication cohort of early-onset AF cases without TTNtv, and underwent CMR. Cardiac volumes and left atrial late gadolinium enhancement (LA LGE), as a fibrosis proxy, were measured by a blinded operator. Results: AF cases with TTNtv had significantly reduced left ventricular ejection fraction (LVEF) compared with controls (57 ± 4 vs 64 ± 5%, P < 0.001). We obtained similar findings in early-onset AF patients without TTNtv compared with controls (61 ± 4 vs 64 ± 5%, P = 0.02). We furthermore found a statistically significant increase in LA LGE when comparing early-onset AF TTNtv cases with controls. Using state-of-the-art CMR, we found that early-onset AF patients, irrespective of TTNtv carrier status, had reduced LVEF, indicating that early-onset AF might not be as benign as previously thought.

## Introduction

Atrial fibrillation (AF) is the most common cardiac arrhythmia. Patients with AF have an increased risk of cardiovascular morbidity, such as stroke and heart failure, and a 1.5–2 fold increased risk of death^1^.

Genome-wide association studies (GWAS) have associated common variants in structural genes, such as *SYNPO2L*^2^ and *TTN*^3^ with AF. Also, rare loss-of-function variants in the *TTN* and *MYL4* gene have recently been linked with early-onset AF^4–7^. With the growing body of evidence implicating structural genes with AF, an “atrial cardiomyopathy” as an underlying substrate for AF has been postulated^8,9^. Patients with an early onset of AF, low CHA_2_DS_2_VASc score and a normal echocardiographic examination are in general considered to have a relatively benign arrhythmia, and current guidelines do not advocate regular clinical assessment^1^. Given the fact that an early debut may indicate a strong heritable component, and that recent evidence suggests impairment in pathways associated with the structural architecture of the cardiomyocyte, it is imperative to explore the clinical course of disease in these early-onset patients^10,11^.

In this study, we aimed to describe the systolic and diastolic function of the left atrium (LA) and ventricle (LV) in patients with early-onset AF and titin-truncating variants (TTNtv). Furthermore, we quantified left atrial late gadolinium enhancement (LA LGE) as a proxy for the degree of fibrosis using cardiac magnetic resonance (CMR).

## Methods

### Study participants

The current study is a clinical follow-up study on a large whole-exome sequencing study of early-onset AF, including familial and non-familial AF patients, and the design has previously been described in detail^4^.

In brief, all individuals with permanent residence in Denmark are assigned a unique civil registration number. Using the civil registration number, we identified all Danish families with three or more family members diagnosed with AF by cross linkage between The National Patient Register and The Danish Family Relations Database. The National Patient Register contains information on all in- and out-of-hospital patient activities in Denmark since 1978^12^. The Danish Family Relations Database contains information on parent-child links in the Danish population and allows for construction of pedigrees^11^. In total, we identified 24 families with at least three members from the same family with an AF registry diagnosis, resulting in 77 familial AF cases available for whole-exome sequencing. A replication cohort of 399 unrelated early-onset non-familial AF patients were also recruited using the Danish National Registries and ICD-10 code I48.9. These were characterized by disease-onset before the age of 40 with normal echocardiographic examination and no traditional risk factors for AF at the time of diagnosis (hyperthyroidism, diabetes, mitral/aortic valve disease, ischemic heart disease, congestive heart failure, congenital heart malformation, or cardiomyopathy).

The familial AF cases had whole-exome sequencing performed and the cohort of early-onset AF patients underwent targeted deep sequencing of *TTN*^4^. Unrelated, early-onset AF patients (familial or non-familial) harboring a TTNtv were offered a CMR. These were matched 1:1 with a cohort of unrelated early-onset AF patients without TTNtv, confirmed by targeted deep sequencing. This was done to evaluate whether findings observed in the TTNtv cohort were generalizable to a non-TTNtv cohort of early-onset AF patients. Matching criteria were gender, age ± 5 years, and body mass index (BMI) ± 1. All early-onset AF patients who were offered CMR, both with and without TTNtv, had disease-onset before the age of 40 with normal echocardiographic examination and no traditional risk factors for AF at the time of diagnosis.

### Control group

We invited a control group for participation through public advertising. Only individuals free of any overt cardiac disease by self-report were included. The control group was matched using the same matching criteria as listed above. Individuals were excluded from the study if they had any general CMR contraindications, i.e. pacemaker, metal implants, claustrophobia, pregnancy, reduced kidney function (plasma-creatinine <60 μmol/l) and previous allergic reactions to the contrast medium gadobutrol.

All participating individuals were given written information and gave written informed consent. The study was approved by the scientific ethics committee for the Capital Region of Denmark (protocol number H-1-2011-044) and complies with the Declaration of Helsinki.

### Image acquisition

CMR scans were performed in a 1.5 Tesla scanner (Aera, Siemens Healthcare, Germany) using an 18-channel body coil. After scout sequences, long axis cine (two-, three- and four-chamber) images were acquired for planning stacks and aid in delineation of chambers. An axial cine stack covering atria and ventricles and a short axis cine stack covering the entire LV were obtained for measurement of LA and LV volumes, respectively (steady-state free precession (SSFP) cine sequences [8 mm; 2 mm gap; 25 phases; field of view (320–360) × 360 adjusted for each patient; matrix size (182–224) × (138–224)] at 10–15 second end-expiratory breath-holds).

LA LGE scans were performed 20 minutes after bolus injection of 0.2 mmol/kg gadobutrol (Gadovist, Bayer, Berlin, Germany), with a maximum of 15 mmol in total. The LA LGE scan consisted of a free-breathing respiration-navigator-gated 3D FLASH sequence with FatSat and ECG-gating (end-atrial diastole, determined from four chamber cine). Typical parameters were: TR/TE 4.67/1.94 ms, bandwidth 300 Hz, inversion time according to scout-sequence (270–310 ms), flip angle 20°, slice thickness 1.5 mm, pixel spacing 0.70 ×0.70 mm and no parallel-imaging.

### Image analyses

CMR analyses were performed in CVI (v. 5.6.6, Circle Cardiovascular Imaging Inc., Calgary, Canada). The images were analyzed by a cardiologist with expertise in CMR who was blinded to participant phenotype and genotype. On short axis cine images, LV endocardial borders were traced manually from end-systolic to end-diastolic phases. Left ventricular outflow tract (LVOT) was included in the blood pool; papillary muscles were excluded, using windowing for the endocardial border. Epicardium was delineated in end-diastolic and end-systolic phases to compare left myocardial mass in end-diastole and end-systole. Left ventricular ejection fraction (LVEF) was defined as abnormal when below 58%^13^.

**Figure 1:**
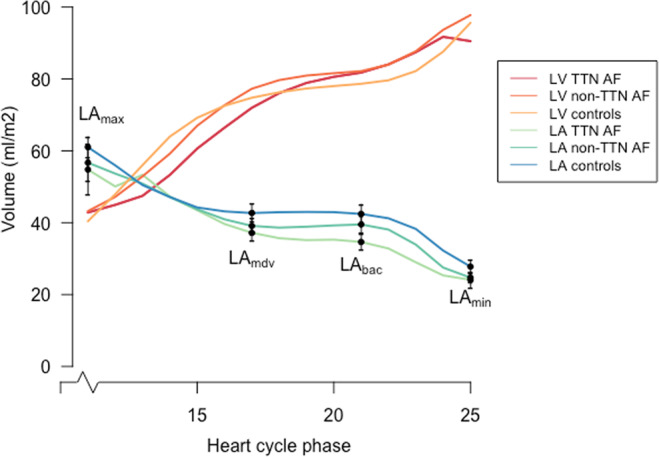
Time-volume-curves. Time-volume curves of the left atrium and left ventricle in early-onset AF cases with TTNtv, early-onset AF without TTNtv and controls. All volumes are indexed to BSA. Shown as mean with error bars representing standard error of the mean. AF, atrial fibrillation; BSA, body surface area; LA, left atrium; LAbac, volume before atrial contraction; LAmax, maximum volume; LAmdv, mid-diastolic volume; LAmin, minimum volume; LV, left ventricle; TTNtv, titin-truncating variants.

LA endocardial borders were traced manually from atrial end-diastolic to end-systolic phases. The pulmonary veins were excluded from the volume and appendages were included. From individual time-volume curves were read atrial volumes corresponding to maximum volume (LA_max_), mid-diastolic volume (LA_mdv_), volume before atrial contraction (LA_bac_), and minimum volume (LA_min_) (see Fig. 1). In participants with AF during CMR we only measured LA_max_, hence no cyclic volumes nor emptying fractions were measured. All volumes were indexed to body surface area. From measured volumes were calculated the following:

LA active emptying volume: LA AEV = LA_bac_ − LA_min_

LA passive emptying volume: LA PEV = LA_max_ − LA_mdv_

LA conduit volume: LA CEV = LVSV – (LA PEV + LA AEV)

LA total emptying fraction: LA TEF = (LA_max_ − LA_min_)/LA_max_ *100

LA passive emptying fraction: LA PEF = (LA_max_ − LA_mdv_)/LA_max_ *100

LA active emptying fraction: LA AEF = (LA_bac_ − LA_min_)/LA_bac_ *100

LA passive/active ratio = (LA PEV/LA AEV) *100

LA LGE scans were analyzed manually off-line. On all axial images, the blood pool as well as the atrial epi- and endocardium were segmented. Pulmonary veins and mitral valves were excluded for fibrosis analyses. All wall intensities above three standard deviations (SD) of the blood pool intensity on every slice were considered enhancement. The total volume and percentage of fibrosis were registered.

### Statistical analyses

Baseline characteristics of the study participants were expressed as number (percentage), mean (SD) or median (interquartile range [IQR]) where appropriate. In the case of non-normality, continuous variables were log transformed.

Student’s t-tests were used to test for differences in LA and LV parameters between the early-onset AF cases with TTNtv compared with the controls and between the early-onset AF replication cohort and the controls. Sensitivity analyses were performed with linear regression models to test the effect of adjustment with antihypertensive medication (beta blockers, aldosterone antagonists, angiotensin-converting-enzyme inhibitors, furosemide and angiotensin II receptor antagonists; yes/no) and heart rate.

We also used Student’s t-test to compare the levels of LA LGE between the study groups. To account for the effect of catheter ablation on cardiac fibrosis^14^, we conducted a subgroup analysis, where individuals were categorized by prior catheter ablation procedure (yes/no). Using this approach, we aimed to isolate the link between fibrosis and AF, without the preponderance of ablation-related fibrosis.

The assumptions of linearity, homoscedasticity and normality were tested graphically using plots of observed versus predicted values and plots of residuals versus predicted values or the observed exposure values. No violations were observed.

A two-sided p-value of less than 0.05 was considered significant. All analyses were conducted using R, version 3.5.3.

## Results

### Clinical characteristics

Baseline characteristics of the study participants are shown in Table 1.

**Table 1:** Baseline characteristics.

	AF with TTNtv (n = 17)	AF without TTNtv (n = 17)	Controls (n = 17)	P-value^†^	P-value^‡^
Gender, male, n (%)	14 (82)	14 (82)	14 (82)	1.0	1.0
Age of AF onset, years (median [IQR])	25 (22–29)	25 (22–29)		—	—
Age at study time (median [IQR])	42 (39–45)	42 (40–45)	42 (37–44)	0.9	0.9
Height, cm (mean [SD]	183 (8)	185 (9)	180 (8)	0.3	0.4
BMI, kg/m^2^ (mean [SD])	26 (4)	27 (4)	26 (3)	0.8	0.6
Smoking, current or previously, n (%)	6 (35)	7 (41)	5 (29)	0.7	0.5
Alcohol consumption per week (mean [SD])	6 (7)	4 (3)	6 (6)	0.8	0.2
Hypertension, n (%)	3 (18)	1 (6)	2 (12)	0.6	0.2
Diabetes, n (%)	0 (0)	1 (6)	0 (0)	—	0.3
Heart failure, n (%)	0 (0)	1 (6)	0 (0)	—	0.3
Myocardial infarction, n (%)	0 (0)	0 (0)	0 (0)	—	—
Type of AF, n (%)					
Paroxysmal	11 (65)	14 (82)		—	—
Persistent	6 (35)	2 (12)		—	—
Permanent	0 (0)	1 (6)		—	—
Family history of AF, self-reported, n (%)	9 (53)	5 (29)		—	—
Previously ablated, n (%)	8 (47)	3 (18)		—	—
Physical exercise level, self-reported, n (%)				0.6	0.7
None	1 (6)	0 (0)	1 (6)		
Mild	5 (29)	8 (47)	9 (53)		
Moderate	9 (53)	7 (41)	6 (35)		
Strenuous	1 (6)	2 (12)	1 (6)		

AF; atrial fibrillation, BMI; body mass index, IQR; interquartile range, NA; not available, SD; standard deviation, TTNtv; titin-truncating variant. ^†^Comparisons between AF patients with TTNtv and controls. ^‡^Comparisons between AF patients without TTNtv and controls.

The study participants were diagnosed with AF at a young age (median age of disease-onset 25 years, IQR 22–29), and had on average been diagnosed with AF for two decades (mean time since AF diagnosis 18 years (± 11 years) at the time of study. Follow-up clinical echocardiographic examinations (median AF duration 9 years, IQR 4–11), revealed normal LVEF and ventricular dimensions in all AF TTNtv patients except one. This patient had suffered from tachycardia-induced heart failure and LA dilation 27 years after diagnosis and now presented with normal LA dimensions and LVEF when subjected to CMR. At the time of study, none of the early-onset AF patients with or without TTNtv had been diagnosed with dilated cardiomyopathy (DCM).

### The left ventricle

In total, 14 early-onset AF TTNtv cases and 17 controls had complete volumetric measurements of LA and LV. Two patients were excluded due to AF during CMR, one patient was excluded due to inability to comply with breath-holding instructions.

Data from comparisons of volumetric measurements of LA and LV are summarized in Table 2 and time-volume curves from the LA and LV are presented in Fig. 1.

**Table 2:** cMRI parameters.

	AF with TTNtv	AF without TTNtv	Controls	P-value^†^	P-value^‡^
Cardiac index, mL/min/m^2^	3856 (601)	3903 (618)	3668 (720)	0.436	0.330
Heart rate, bpm	72 (12)	66 (10)	62 (13)	0.018^§^	0.264
LA volumes, ml/m^2^					
LAmin_i_	24 (7)	25 (5)	27 (8)	0.252	0.232
LAmax_i_	52 (12)	55 (10)	60 (11)	0.103	0.264
LAmdv_i_	34 (8)	37 (8)	41 (10)	0.059	0.317
LAbac_i_	35 (9)	40 (9)	44 (11)	0.025^§^	0.278
LA emptying volumes, ml/m^2^					
LA passive emptying volume_i_	18 (6)	18 (5)	19 (6)	0.705	0.625
LA active emptying volume_i_	11 (4)	15 (5)	16 (4)	0.002^§^	0.530
LA conduit volume_i_	25 (5)	26 (6)	25 (6)	0.779	0.432
LA total emptying fraction	54 (9)	56 (6)	55 (6)	0.793	0.571
LA passive emptying fraction	34 (6)	33 (7)	32 (9)	0.456	0.808
LA active emptying fraction	32 (9)	38 (7)	37 (5)	0.059	0.817
LA passive/active ratio	168 (2)	122 (2)	115 (2)	0.032^§^	0.739
LA LGE, %	8 (2)	5 (2)	3 (3)	<0.001^§^	0.064
LV parameters					
LV mass, g/m^2^	62 (11)	63 (9)	65 (12)	0.408	0.542
LVEF, %	57 (4)	61 (4)	64 (5)	<0.001^§^	0.020^§^
LVEDV, ml/m^2^	95 (17)	98 (10)	94 (15)	0.767	0.272
LVESV, ml/m^2^	41 (7)	39 (5)	34 (8)	0.013^§^	0.033^§^
LV stroke volume, ml/m^2^	53 (1)	59 (1)	59 (1)	0.174	0.980
LVPFR, ml/s/m^2^	266 (59)	296 (79)	316 (74)	0.049	0.472

Values are shown as mean (SD) unless otherwise stated. All volumes are indexed to BSA (marked with a subscript i). Individuals with complete volumetric measurements: AF with TTNtv; n = 14, AF without TTNtv; n = 15, controls; n = 17. Individuals with complete LA LGE measurements: AF with TTNtv; n = 17, AF without TTNtv; n = 17, controls; n = 13. AF, atrial fibrillation; bpm, beats per minute; BSA, body surface area; LA, left atrial; LA_bac_, LA volume before atrial contraction; LA_mdv_, LA mid-diastolic volume (volume after passive emptying but before mid-diastolic expansion); LA LGE, left atrial late gadolinium enhancement; LV, left ventricular; LVEF, LV ejection fraction; LVEDV, LV end-diastolic volume; LVESV, LV end-systolic volume; LVPFR, LV peak filling rate; SE, standard error; TTNtv, titin-truncating variant. ^†^Comparisons between AF patients with TTNtv and controls. ^‡^Comparisons between AF patients without TTNtv and controls.

^§^Statistically significant.

When examining the LV parameters, we found significantly reduced LVEF (57 ± 4 vs 64 ± 5%, P < 0.001), due to higher LV end-systolic volume (LVESV; 41 ± 7 vs 34 ± 8 ml/m^2^, P = 0.013) in early-onset AF TTNtv cases compared with controls. We found no statistically significant difference in LV end-diastolic volume (Table 2). To validate these findings, we investigated the same volumetric parameters in a replication cohort of early-onset AF patients without TTNtv. In total, 15 early-onset AF patients had complete volumetric measurements of LA and LV. Two patients were excluded due to AF during CMR. We found similar trends, albeit a smaller reduction in LVEF (61 ± 4 vs 64 ± 5%, P = 0.020) and increase in LVESV (39 ± 5 vs 34 ± 8 ml/m^2^, P = 0.033) in early-onset AF patients without TTNtv compared with controls. The results did not materially change when adjusting for antihypertensive medication or heart rate. However, LVESV was no longer statistically significant when comparing early-onset AF patients without TTNtv with controls and adjusting for antihypertensive medication.

### The left atrium

When examining the LA volumetric measurements, the mid-cyclic volume LA_bac_ was significantly reduced in early-onset AF patients with TTNtv compared with controls (35 ± 9 vs 44 ± 11 ml/m^2^, P = 0.025). The lower mid-cyclic volume could also be seen in a reduced LA active emptying volume (11 ± 4 vs 16 ± 4 ml/m^2^, P = 0.002), whereas we found no significant difference in LA passive emptying volumes between groups. The reduction in the actively emptied volume could also be seen in a significantly higher LA passive/active ratio in early-onset AF TTNtv cases compared with controls (168 ± 2 vs 115 ± 2, P = 0.032) (Table 2). We found no significant differences in LA volume indices between early-onset AF patients without TTNtv and controls. When adjusting for antihypertensive medication, the other mid-cyclic volume LA_mdv_ also became significantly different between early-onset AF TTNtv cases and controls (mean difference −7 ml/m^2^, 95% confidence interval −14; −1, P = 0.032). Adjustment for antihypertensive medication did not alter the remaining comparisons. When adjusting for heart rate, LA_bac_ was no longer significantly different between early-onset AF TTNtv cases and controls.

### LA LGE measurements

A total of 17 early-onset AF TTNtv cases and 13 controls had complete LA LGE CMR scans performed. Four controls were excluded due to low quality of the LA LGE scan.

We found a significant increase in the level of LA LGE (8 ± 2 vs 3 ± 3%, P < 0.001) when comparing early-onset AF TTNtv cases with controls. This finding could not be replicated in early-onset AF patients without TTNtv compared with controls.

**Figure 2:**
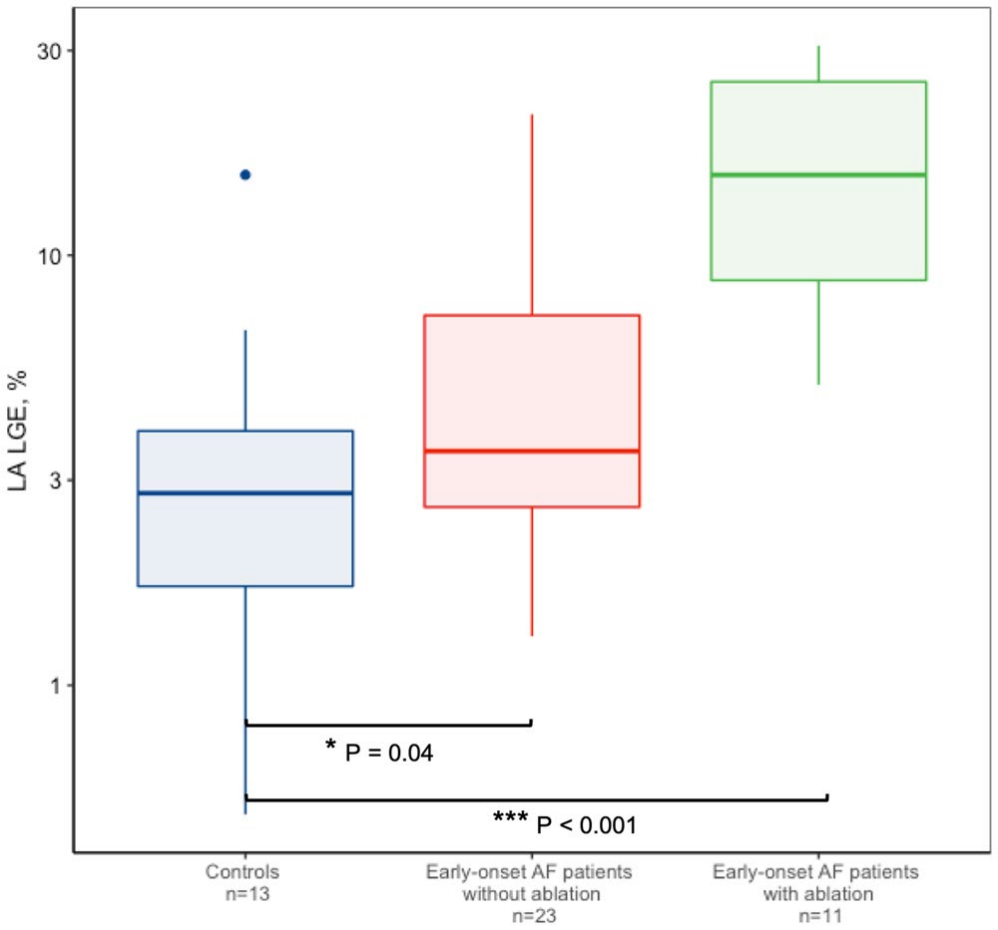
Left atrial late gadolinium enhancement in all ablated/non-ablated early-onset AF patients. Median left atrial late gadolinium enhancement values and interquartile range for controls (n = 13), early-onset AF patients without (n = 23) and with (n = 11) ablation, respectively. Y-axis on logarithmic scale. AF, atrial fibrillation.

To investigate whether the difference in LA LGE levels was solely caused by previous ablation therapy procedures, we furthermore compared all non-ablated and ablated early-onset AF patients with controls. We found a significant increase in LA LGE in non-ablated early-onset AF patients compared with controls (4 ± 2 vs 3 ± 3%, P = 0.042), and, as anticipated, in ablated early-onset AF patients compared with controls (14 ± 2 vs 3 ± 3%, P < 0.001) (Fig. 2).

**Figure 3:**
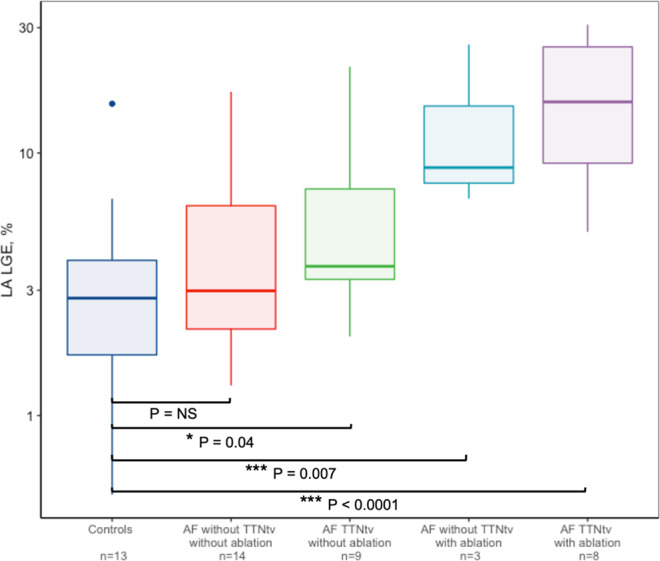
Left atrial late gadolinium enhancement in ablated/non-ablated early-onset AF patients with and without TTNtv. Median left atrial late gadolinium enhancement values and interquartile range for controls (n = 13), AF patients without TTNtv with (n = 3) and without (n = 14) previous ablation and AF patients with TTNtv with (n = 8) and without (n = 9) previous ablation procedures. Y-axis on logarithmic scale. AF, atrial fibrillation; TTNtv, titin-truncating variants.

Comparisons of LA LGE levels in ablated/non-ablated early-onset AF patients with and without TTNtv can be found in Fig. 3.

Overall, when evaluating the spatial distribution of LA LGE on MRI scans through eye-balling, we found no significant LA LGE in the controls, whereas early-onset AF TTNtv cases without previous ablation had diffusely dispersed LA LGE, as exemplified in Supplemental Video 1 and 2 and Supplementary Figure 1.

## Discussion

In this study, we investigated the relationship between early-onset AF patients with and without TTNtv and dynamic parameters of the left atrium and ventricle and the amount of fibrosis in the atrial wall, using state-of-the-art cardiac imaging.

### Reduced LVEF in early-onset AF patients

We found that early-onset AF patients with TTNtv had a discrete, but statistically significant reduction in LVEF compared with controls. TTNtv have previously been associated with cardiomyopathies^15^ and previous studies have shown that DCM patients with TTNtv have increased ventricular interstitial fibrosis and an overall higher rate of ventricular arrhythmias^16^. In the present study, none of the CMR investigated early-onset AF cases had been diagnosed with DCM at the time of study, and echocardiographic examinations up to 40 years after diagnosis had been normal in 16 out of 17 patients. Consistent with our results, Haggerty *et al*. found that healthy individuals with TTNtv and without a cardiomyopathy diagnosis have a decreased LVEF evaluated by echocardiographic examinations, although more modest compared with individuals with TTNtv and cardiomyopathy^17^. We also found a similar, modest LVEF reduction, in our cohort of early-onset AF patients without TTNtv. This may partly be explained by the established link between AF and heart failure, or that early-onset AF patients without TTNtv may harbor other structural genetic defects that may induce a comparable (albeit more moderate) phenotype as those with TTNtv, compared with older adults with a more traditional onset of AF. Of note, the LVEF reference normal value based on SSFP CMR is between 58 and 76% for healthy 40–49 year old individuals^13^, and a reduced LVEF, even in the low normal range, has been associated with an increased risk of congestive heart failure^18^. Early-onset AF is in general considered a benign arrhythmia, and regular clinical assessment, in particular with regards to the functional properties of the heart, is not standard. From a clinical perspective, despite that patients with an early presentation of disease may have an overall low thromboembolic risk, our study indicates that an early debut of AF may represent an early marker for an increased risk of a prematurely failing heart. In this regard, early genetic testing may be warranted to identify patients at particularly high risk (TTNtv carrier status), in order to discern carrier status and identify those who might benefit from early clinical assessment, and closer follow-up.

### Early-onset atrial fibrillation and atrial indices

With regards to the LA measurements, the most noticeable difference between the groups was found in the active emptying volume and LA LGE. Compared to controls, the active emptying volume was significantly reduced in early-onset AF patients with TTNtv, illustrated in the time-volume curves drawn from the volumetric measurements (Fig. 1). This indicates that reductions in atrial active emptying could represent a marker for atrial disease whereas atrial passive emptying is correlated to LV diastolic dysfunction and found not to be indicative of AF, as previously shown^19^. Kojima *et al*. found that atrial function was impaired prior to LA enlargement and that this was especially evident for the active function^20^. In fact, we found higher means of LA_max_ and LA_min_ in the control group compared with the two early-onset AF groups. This may be due to a higher heart rate in the AF patients and possibly due to the fact that many of the control subjects were accustomed to moderate or strenuous exercise.

We also identified a significant difference in the amount of LA LGE in early-onset AF cases with TTNtv compared with controls. Early-onset AF patients without TTNtv did not have a significant increase in LA LGE. We also found that these differences were not mediated through higher ablation rates in patients with early-onset AF (Fig. 2). These findings could indicate that early-onset AF patients in general have an increased level of fibrosis in the atrial wall. LA LGE has previously been correlated with active atrial function measured with strain^21^, indicating that active function and LA LGE are both associated with atrial disease, possibly because they are markers of the remodeled LA.

Structural cardiac genes have within recent years been associated with AF, including the sarcomeric protein gene myosin light chain 4 (*MYL4*)^6,7,22^, *SYNPO2L*^2,23^, and lately the *TTN* gene^3^ encoding the giant sarcomeric protein titin highly expressed in all four heart chambers.

An increase in atrial fibrosis in AF patients compared with non-AF patients^24–27^ and in lone AF patients compared with non-lone AF patients^28^ has previously been reported. Kottkamp *et al*.^26^ suggested to reclassify some lone AF patients as having “fibrotic atrial cardiomyopathy”. Wilson *et al*. investigated atrial fibrosis in high-risk AF pedigrees and found these to have elevated levels, which could indicate that a heritable contribution of atrial fibrosis leads to a predisposition to AF^29^. The relationship between atrial fibrosis and AF is, however, complex, and further functional studies are needed to establish whether there is a causal link between levels of fibrosis and AF.

## Limitations

The average heart rate was higher in the AF patients (with and without TTNtv) compared with the controls. This may have had some impact on LA and LV volumes and function, however not to the extent that we found^30^.

The LA LGE method has several limitations. Due to a genotype-driven study setup, some AF patients both with and without TTNtv had been subjected to ablation therapy, which of course influence the LA LGE measurements. The atrial wall is between 1–4 mm, which often challenges the spatial resolution of the 3D sequence. Thus, difficulties in delineation of non-enhanced wall in general includes the risk of also including blood volume and epicardial tissue.

Time from early-onset AF diagnosis to CMR scan was on average nearly 20 years. We therefore cannot rule out that some of the changes seen are caused by other factors. The lack of significant associations in some comparisons could be caused by the modest study size and the results could benefit from replication in larger sample sizes.

## Conclusions

In conclusion, we found evidence of an, albeit subclinical, affected LV systolic function and increased amounts of atrial fibrosis in early-onset AF patients. These results indicate that some early-onset AF patients might benefit from early genetic testing and clinical evaluation, in order to establish who should be considered for regular clinical assessment.

## Supplementary information

Supplementary information.

Supplementary information 2.

Supplementary information 3.

## Data Availability

The datasets generated and/or analyzed during the current study are available from the corresponding author on reasonable request.
