# Supplementary material for: Early-onset atrial fibrillation patients show reduced left ventricular ejection fraction and increased atrial fibrosis
Source: Sci Rep. 2020 Jun 22;10:10039. doi: 10.1038/s41598-020-66671-w (PMC7308347; doi:10.1038/s41598-020-66671-w)
Supplement: Supplementary file 1 — Supplementary information. [file 41598_2020_66671_MOESM1_ESM.docx]

**Supplementary Material**

**Early-onset atrial fibrillation patients show reduced left ventricular ejection fraction and increased atrial fibrosis**

Laura Andreasen^a,b,#,*^, MD, Litten Bertelsen^c,#^, MD, Jonas Ghouse^a,b^, MD, Pia R. Lundegaard^a,b^, MSc, PhD, Gustav Ahlberg^a,b^, MSc, Lena Refsgaard^a,b^, MD, PhD, Torsten B. Rasmussen, MD, PhD^d^, Hans Eiskjær, MD, DMSc^d^, Stig Haunsø^a,e^, MD, DMSc, Niels Vejlstrup^c^, MD, PhD, Jesper H. Svendsen^c,e^, MD DMSc, Morten S. Olesen^a,b^, MSc, PhD.

The work was carried out at the Department of Cardiology, Rigshospitalet, and the Department of Biomedical Sciences, University of Copenhagen, Copenhagen, Denmark.

^a^Laboratory for Molecular Cardiology, Department of Cardiology, Centre for Cardiac, Vascular, Pulmonary and Infectious Diseases, Rigshospitalet, University Hospital of Copenhagen, Copenhagen, Denmark.

^b^Department of Biomedical Sciences, University of Copenhagen, Copenhagen, Denmark.

^c^Department of Cardiology, Centre for Cardiac, Vascular, Pulmonary and Infectious Diseases, Rigshospitalet, University Hospital of Copenhagen, Copenhagen, Denmark.

^d^Department of Cardiology, Aarhus University Hospital, Aarhus, Denmark

^e^Department of Clinical Medicine, University of Copenhagen, Copenhagen, Denmark.

^#^These authors contributed equally to this work.

**Table of Contents**

[Supplementary Video 1 and 2 3](#_Toc40106358)

[Supplementary Figure 1 4](#_Toc40106359)

# **Supplementary Video 1 and 2**

Video sequence of long axis cine images from CMR scans after gadobutrol injection. Video 1) control, video 2) non-ablated atrial fibrillation patient with titin-truncating variant. Signal enhancement indicates increased levels of LA late gadolinium enhancement as a proxy for fibrosis.
Ao, aorta; CMR, cardiac magnetic resonance imaging; LA, left atrium; LV, left ventricle.

# **Supplementary Figure 1**


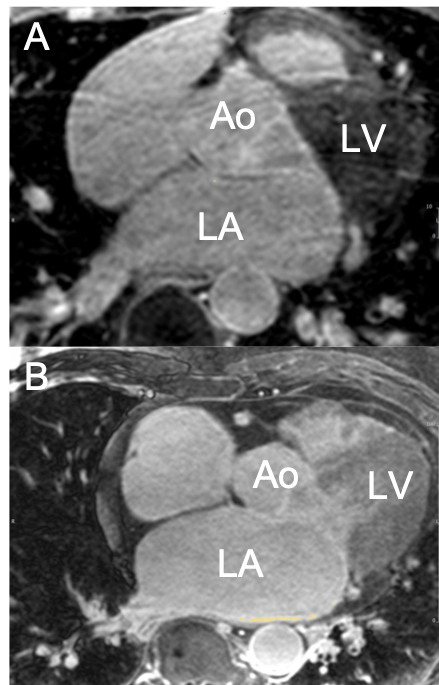


**Still image from Supplementary Video 1 and 2.** A) Still image from control, and B) non-ablated atrial fibrillation patient with titin-truncating variant. Areas with intensities more than 3SD above blood pool indicating late gadolinium enhancement as a proxy for fibrosis are marked with yellow.

Ao, aorta; LA, left atrium; LV, left ventricle.
